# A Preliminary Study Showing the Impact of Genetic and Dietary Factors on GC–MS-Based Plasma Metabolome of Patients with and without *PROX1*-Genetic Predisposition to T2DM up to 5 Years Prior to Prediabetes Appearance

**DOI:** 10.3390/cimb43020039

**Published:** 2021-06-29

**Authors:** Patrycja Mojsak, Katarzyna Miniewska, Adrian Godlewski, Edyta Adamska-Patruno, Paulina Samczuk, Fernanda Rey-Stolle, Witold Bauer, Coral Barbas, Adam Kretowski, Michal Ciborowski

**Affiliations:** 1 Clinical Research Centre, Medical University of Bialystok, Sklodowskiej-Curie 24A, 15-276 Bialystok, Poland; patrycja.mojsak@umb.edu.pl (P.M.); katarzyna.miniewska@umb.edu.pl (K.M.); adrian.godlewski@umb.edu.pl (A.G.); edyta.adamska@umb.edu.pl (E.A.-P.); paulina.samczuk@umb.edu.pl (P.S.); witold.bauer@umb.edu.pl (W.B.); adam.kretowski@umb.edu.pl (A.K.); 2 Centre for Metabolomics and Bioanalysis (CEMBIO), Department of Chemistry and Biochemistry, Facultad de Farmacia, Universidad San Pablo-CEU, CEU Universities, Urbanización Montepríncipe, Boadilla del Monte, 28660 Madrid, Spain; frstolle@ceu.es (F.R.-S.); cbarbas@ceu.es (C.B.); 3 Department of Endocrinology, Diabetology and Internal Medicine, Medical University of Bialystok, Sklodowskiej-Curie 24A, 15-276 Bialystok, Poland

**Keywords:** NC/HC-meal intake, *PROX1*, plasma, GC–MS, metabolomics, prediabetes, T2DM, carbohydrates, amino acids, fatty acids

## Abstract

Risk factors for type 2 diabetes mellitus (T2DM) consist of a combination of an unhealthy, imbalanced diet and genetic factors that may interact with each other. Single nucleotide polymorphism (SNP) in the prospero homeobox 1 (*PROX1*) gene is a strong genetic susceptibility factor for this metabolic disorder and impaired β-cell function. As the role of this gene in T2DM development remains unclear, novel approaches are needed to advance the understanding of the mechanisms of T2DM development. Therefore, in this study, for the first time, postprandial changes in plasma metabolites were analysed by GC–MS in nondiabetic men with different *PROX1* genotypes up to 5 years prior to prediabetes appearance. Eighteen contestants (12 with high risk (HR) and 6 with low risk (LR) genotype) participated in high-carbohydrate (HC) and normo-carbohydrate (NC) meal-challenge tests. Our study concluded that both meal-challenge tests provoked changes in 15 plasma metabolites (amino acids, carbohydrates, fatty acids and others) in HR, but not LR genotype carriers. Postprandial changes in the levels of some of the detected metabolites may be a source of potential specific early disturbances possibly associated with the future development of T2DM. Thus, accurate determination of these metabolites can be important for the early diagnosis of this metabolic disease.

## 1. Introduction

Type 2 diabetes mellitus (T2DM) is a complex polygenic disorder [1] characterised by the incapability of pancreatic β-ells to increase insulin secretion to compensate for insulin resistance (IR) in peripheral tissues [2]. According to the latest epidemiological studies, the prevalence of people with T2DM worldwide was 463 million in 2019, and this number is expected to increase to 592 million by 2035 [3] and 629 million by 2045 [2]. However, rapid implementation of appropriate prevention and treatment strategies is challenging due to problems with the diagnosis of T2DM in its early stages. The main reason is that the symptoms of T2DM are not obvious or only partially manifested at the beginning of the disease [4]. Additionally, conventional clinical and blood biomarkers, such as BMI, fasting blood glucose or HbA1c levels, are well-established predictors but remain imperfect and provide limited insight regarding underlying pathophysiology [5].

It was confirmed that risk factors for T2DM consist of a combination of an imbalanced diet, sedentary lifestyle and genetic factors that may interact with each other [1]. A proper diet and systematic physical activity are essential lifestyle determinants of potential T2DM development. However, the role of these modifiable factors in the prevention of T2DM strongly depends on genetic susceptibility. Therefore, an indication of T2DM risk subgroups with genetic characteristics known to promote disease development, especially sensitive to specific foods, nutrients or physical activity, may help design and implement individualised and targeted intervention and/or prevention strategies [6]. We have been progressing our understanding of the genetic susceptibility to T2DM, including single-nucleotide polymorphisms (SNPs) of particular genes. Several genes have been identified that may be associated with T2DM; among these, the prospero homeobox 1 (*PROX1*) is considered an important gene for T2DM risk due to its function regulating glucose-induced insulin secretion [7]. Already, in the year 2005, Harvey et al. [8] reported an animal model in which *PROX*1 heterozygous adult mice became obese and had higher serum insulin levels and hepatic lipid accumulation. In the study conducted by Kretowski et al. [9], it was reported that people who possess this variant are more glucose intolerant and have more visceral fat than people lacking it. It was also indicated that mechanisms by which the *PROX1* gene affects the susceptibility to T2DM seem to be more complex [9]. Thus, identifying individuals with high risk for T2DM and elucidating the underlying mechanisms is crucial for developing effective strategies to prevent this disease [5].

The surge in the prevalence of T2DM during the past several decades cannot be explained by only genetic factor alone. It was confirmed that inappropriate diet combined with a genetic predisposition might be a factor accelerating specific changes in the metabolic profile of people susceptible to T2DM. However, reproducible data supporting gene–diet interaction are still sparse, and little knowledge about gene–diet interactions has been applied in public health practice. Thus far, only a few studies have indicated interactions between specific dietary components and individual genetic variants [1,10,11]. Yet, some findings have been replicated, and it is unclear whether the importance of overall dietary habits, including T2DM-related food intake, differs depending on the overall genetic profile [1]. Our recent study [11] was the first to study the influence of *PROX1* gene–diet interaction on the plasma metabolome of healthy genetic risk carriers. In this study, liquid chromatography quadrupole time-of-flight mass spectrometry (LCQTOF-MS) was used to evaluate postprandial changes in plasma metabolites during the high-carbohydrate (HC) and normo-carbohydrate (NC) meal-challenge tests in nondiabetic men with different polymorphisms in the *PROX1* rs340874 gene. This study revealed that plasma metabolites postprandially changing in the high-risk *PROX1* genotype carriers belong to T2DM-related metabolic pathways [11].

Metabolomics has already allowed the identification of metabolites that can serve as potential biomarkers for the diagnosis or treatment of T2DM [12]. The relationships between metabolites level and insulin resistance [13,14], prediabetes [15,16] and T2DM [4,17] have been evaluated in several studies using mass spectrometry (MS) coupled with gas (GC) or liquid (LC) chromatography. Thus far, metabolites such as aromatic amino acids (AAAs), branched-chain amino acids (BCAAs), sugar metabolites and gluconeogenesis substrates (including glucose and fructose), and finally, different lipid subclasses (such as phospholipids, sphingomyelins, triglycerides and also specific lipids), identified using high-throughput metabolomics, have been associated with T2DM or different stages of its development. Considering metabolomics research, LC–MS and GC–MS are complementary platforms [5], and reviewed literature indicate that many T2DM-related plasma or serum metabolites can be measured using GC–MS [18,19,20]. Additionally, GC–MS is one of the most efficient, reproducible and convenient methods for quantitative and comprehensive metabolomics analysis due to its robustness, excellent separation capability, selectivity and sensitivity [19].

Genetic susceptibility to type 2 diabetes includes single-nucleotide polymorphisms (SNPs) of several genes. Prospero-related homeobox 1 (*PROX1*) plays pivotal roles in the embryonic formation of several organs and tissues, including liver, pancreas, eye, lymphatic vessel, nerve and cardiac muscle in mice [1,2,3,4] and a meta-analysis of a genome-wide association study revealed that the rs340874 SNP in the *PROX1* gene is associated with type 2 diabetes reported in an animal model in which *PROX1* heterozygous adult mice become obese and had higher serum insulin levels and hepatic lipid accumulation.

The present study is a continuation of the above-mentioned study of Adamska-Patruno et al. [11]. To complement previous meal-challenge metabolomics results, in the present work, metabolites were measured in samples from the same patients using GC–MS. Of note, participants of this study were recruited from the cross-sectional study called 1000 PLUS, which has been described in details previously [21]. We have recently performed 5-years of follow-up visits with the individuals from the 1000 PLUS cohort [22]. Interestingly, half of the risk carriers from the present study participated in the follow-up visit, and we observed that their parameters assessing glucose homeostasis (e.g., fasting plasma glucose, HOMA-IR, HbA1c) have worsened, indicating the development of a prediabetic state. Therefore, obtained results revealed novel meal-affected metabolites, which may be connected with the process of T2DM development within the next 5 years. Consequently, this study is of great importance, as it provides new insights into *PROX1* gene–diet interactions and potential T2DM development.

## 2. Materials and Methods

### 2.1. Samples

The study was conducted on selected Polish-origin Caucasian volunteers recruited to the meal-test study from the previously described [11,21] 1000 PLUS cohort. This trial was registered at www.clinicaltrials.gov (accessed on 15 April 2021) as NCT03792685. Taking into consideration the fact that investigated factors can be characterised by sexual dimorphism [23], only male participants were included in the study group. Participants (n = 18) were divided into 2 groups based on the *PROX1* rs340874 genotypes: the homozygous carriers of high-risk (HR) allele C (CC genotype, n = 12) and carriers of low-risk (LR) allele T (TT genotypes, n = 6). When the meal-challenge study was conducted, all participants did not suffer from T2DM, prediabetes or other disorders and did not receive any treatment that might affect the results. Based on the daily physical activity measure assessed with the use of self-administered questionnaires [24], all participants were classified as having moderate or high (the majority of the participants) physical activity. A daily energy intake was also similar for all participants (1991.8 ± 529.7 kcal) with 20.4% ± 4.4% (19.4% ± 1.4% vs. 21.6% ± 4.3% for LR vs. HR, *p* = 0.4) of energy from protein, 33.6% ± 5.8% (33.9% ± 10.0% vs. 31.7% ± 5.3% for LR vs. HR, *p* = 0.9) from fat and 42.0% ± 6.3% (41.4% ± 6.2% vs. 42.7% ± 6.8% for LR vs. HR, *p* = 0.9) from carbohydrates. Five years after the first examination, subjects from the 1000 PLUS cohort were called for a follow-up visit [22]. Seven individuals from the risk carriers group responded to this call, and for six of them, we observed worsening of selected clinical parameters assessing glucose homeostasis (e.g., fasting plasma glucose, HOMA-IR, HbA1c), indicating the development of prediabetic state manifested by increased (within the range of 100–125 mg/dL) fasting plasma glucose (three individuals) or increased (within the range of 5.7–6.4%) HbA1c value (two individuals) or insulin resistance (HOMA-IR = 4.6, one individual).

### 2.2. Regents

Pentadecanoic acid (99%), methyl stearate standards, HPLC grade methanol and silylation-grade pyridine were purchased from Sigma-Aldrich (Steinheim, Germany). Reagents for derivatisation (O-methoxyamine hydrochlorideand BSTFA:TMCS (N,O-Bis(trimethylsilyl)trifluoroacetamide with 1% Trimethylchlorosilane), 99:1 (SylonBFT)) were purchased from Sigma-Aldrich (Steinheim, Germany) and Supelco (Belle-fonte, PA, USA), respectively. Two standard mixes for GC–MS, one containing grain fatty acid methyl esters (C8:0–C22:1, n9) and another standard mix with a mixture of n-alkanes (C8–C40) and analytical grade heptane, were purchased from Fluka Analytical (Sigma-Aldrich Chemie GmbH, Steinheim, Germany). 

### 2.3. Working Solution and Standards

Individual stock solutions of 4-nitrobenzoic acid (4-NBA) and methyl stearate (IS) were prepared in methanol and stored at −20 °C. These solutions were used to prepare an intermediate solution of each compound which were stored at 4 °C during the working week and appropriately diluted on the day of the analysis.

### 2.4. Ethics

The study procedures were conducted in accordance with all of the ethical standards of human experimentation and with the Declaration of Helsinki. The study protocol was approved by the local Ethics Committee (Medical University of Bialystok, Poland, R-I-002/35/2009), and before any study procedures, all the participants signed informed consent.

### 2.5. Meal Challenge Tests

The volunteers were studied twice, within an interval of 2 to 3 weeks, in random order. Participants were instructed to avoid coffee, alcohol and excessive physical exercise on the day before each test and to maintain their regular lifestyle throughout the study. After fasting blood collection, participants received a standardised HC-meal (300 mL, Nutridrink Juice Style, Fat Free, Nutricia, Poland), which provided 450 kcal (89% of energy from carbohydrate—around 100 g, 11% from protein—around 12 g and 0% from fat, or an NC-meal (360 mL, Cubitan, Nutricia, Poland), providing 450 kcal (45% of energy from carbohydrate—around 50 g, 30% from protein-around 34 g and 25% from fat—around 13 g, choline 69 mg/100 mL). The blood for metabolomics analyses was collected at fasting state and 30, 60 and 120 min after meal intake.

### 2.6. Metabolomics Analysis

Metabolite extraction was performed as described by Dudzik et al. [25] with a small modification. Plasma (40 μL) was deproteinised with 120 μL of 25 ppm 4-NBA in cold acetonitrile (1:3, −20 °C), followed by two-step derivatisation: (i) methoximation with O-methoxyamine hydrochloride in pyridine (15 mg/mL, room temperature, 16 h) followed by (ii) silylation with BSTFA containing 1% TMCS (70 °C, 1 h). Metabolic fingerprinting (MF) was performed using an HP 6890 Series GC system equipped with an HP 6890 autosampler and a Mass Selective Detector 5973 (Agilent Technologies, Palo Alto, CA, USA). 1 μL of the derivatised plasma sample with IS was injected onto a DB-5MS capillary GC column (30 m × 0.25 mm × 0.25 μm) using helium as carrier gas at a constant gas flow of 1.0 mL/min. The injector temperature was set at 250 °C and the split ratio to 1:10. The temperature gradient program started at 60 °C held for 1 min, followed by a subsequent increase in temperature to 320 °C at a rate of 10 °C/min. The GC–MS transfer line, filament source and the quadrupole temperature were set at 280, 230 and 150 °C, respectively. The electron ionisation (EI) source was set at 70 eV, and the mass spectrometer was operated in full scan mode, applying a mass range from *m*/*z* 50 to 600 at a scan rate of 1.38 scan/s.

### 2.7. Quality Control Samples

To determine the reproducibility of plasma sample preparation and the stability of the analytical platform used, several QC samples were prepared by mixing equal volumes of all analysed samples. Subsequently, preparation of the QC samples was performed using the same procedures as was described above. QC runs were performed prior to the analysis of all plasma samples until system equilibration was achieved.

### 2.8. GC–MS Data Treatment

Total ion chromatograms (TICs) obtained after the analysis were inspected based on both, quality of the chromatograms and internal standard signals. At first, samples were processed with Mass Hunter Workstation GC/MS Translator software (version B.04.01) in order to make them compatible with the Mass Hunter Quantitative data analysis (version B.08.00). The resulting data files were exported to Agilent Mass Hunter Unknowns Analysis Tool (version 7.0) for the deconvolution process and metabolites’ identification based on GC–MS raw data. In order to obtain a chemical identity of the compounds, the software executed a search against two target libraries. Fiehn’s library (version 2013) was used to compare retention time (RT) and spectra extracted during the deconvolution against each compound included in the library. For the non-identified compounds, a mixture of n-alkanes, that was analysed at the beginning of the analytical run was used as a reference for retention time and retention index (RI) comparison with a commercial NIST (National Institute of Standards and Technology) spectral library (version 2.2, 2019). Metabolites with spectrum score higher than 80% and concordant RI based on the alkanes scale were putatively identified using the NIST library. Data obtained by the Unknown Analysis Tool were aligned using Mass Profiler Professional B.12.1 (Agilent Technologies, Santa Clara, CA, USA). Then, Agilent Mass Hunter Quantitative Analysis (version B.08.00) was used for the assignment of the target and qualifiers ions and peak area integration. Prior to the statistical analysis, sample areas were normalised by methyl stearate abundance in order to minimise the response variability coming from the instrument. Finally, data were filtered based on the coefficient of signal variation (CV) in QC samples, considering values lower than 30% as acceptable.

### 2.9. Pathway Analysis with MetaboAnalyst

Metabolic pathway analysis was performed to identify clusters of metabolites related to key cellular signalling and metabolic networks, which may provide mechanistic insight into the underlying biology of differentially expressed metabolites. For this purpose, MetaboAnalyst 5.0 (http://www.metaboanalyst.ca, accessed on 5 June 2021) was used, and pathway analysis was performed for statistically significant metabolites detected in this and our previous [11] study. To increase the specificity of the results, *Homo sapiens* organism was selected in the KEGG database.

### 2.10. Statistical Analysis

Statistical analysis was performed for NC- and HC-meal independently. Due to the small number of contestants in each group, non-parametric tests were chosen. The Wilcoxon signed-rank test was performed to study the differences between metabolites level in dependent samples (the level of the metabolite in 30′, 60′ and 120′ after meal intake compared to their fasting level), whereas the Mann–Whitney U test was used to test the differences between the level of metabolites in the fasting state of HR- and LR-genotype groups. Statistical analysis was performed using the R software environment (version 4.0.0, https://www.R-project.org/, accessed on 15 February 2021).

## 3. Results

### 3.1. Baseline Characteristics

The baseline characteristics of the studied population are presented in Table 1. The groups were well matched without any between-group differences in age, anthropometric measurements (body mass index (BMI), body fat and fat-free mass content), fasting glucose and insulin level, as well as HOMA-IR, HOMA-B and glycated haemoglobin (HbA1c).

### 3.2. Metabolomics Results

A GC–MS-based approach was applied for plasma metabolomics analysis of plasma samples from 18 patients. Considering the fact that the run time per sample is 37.5 min, samples were analysed in two analytical batches, according to the type of meal taken (NC- and HC-meal).

**Figure 1:**
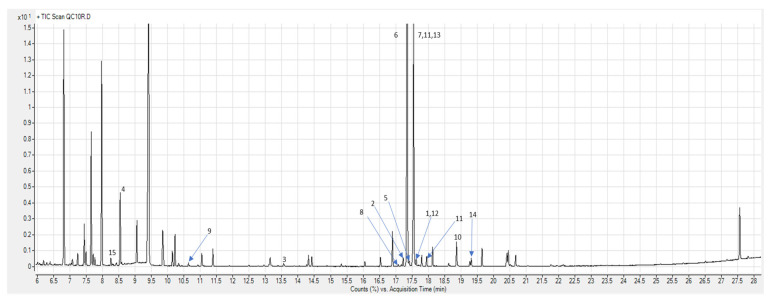
Total ion chromatogram (TIC) of plasma profile obtained by GC–MS with marked statistically significant metabolites. 1. Alanine, 2. Histidine, 3. Creatinine, 4. Norleucine, 5. Galactosamine, 6. Galactose, 7. Allose, 8. Fructose, 9. Glyceric acid, 10. Palmitic acid, 11. 5-Keto-D-gluconate, 12. Gluconic acid, 13. Tyramine, 14. Uric acid, 15. α-Hydroxybutyric acid.

First, 525 and 624 raw peaks after the NC-meal and HC-meal were observed in GC–MS data, respectively. After data pretreatment (deconvolution, alignment, data normalisation and filtering), 125 entities were obtained. Finally, 58 metabolites with CVs below 30% in the QC samples of HC- or NC-meal analyses were annotated. In total, 15 significantly different (*p* < 0.05) metabolites were identified (Figure 1). Similarly to our previous study [11], at the fasting state, we did not observe any significant differences in metabolites’ levels between studied groups. Whilst postprandially, the HR genotype carriers presented differences in the level of 11 and 5 metabolites after HC- and NC-meal intake, respectively. In the case of LR genotype carriers, we have not observed any metabolites significantly changing postprandially. In the case of LR genotype carriers, we did not observed any metabolites significantly changing postprandially. Metabolites significant in the *PROX1* low- (LR) and high-risk (HR) genotype men after HC-meal and NC-meal intake are presented in Table 2 and Table 3, respectively.

**Figure 2:**
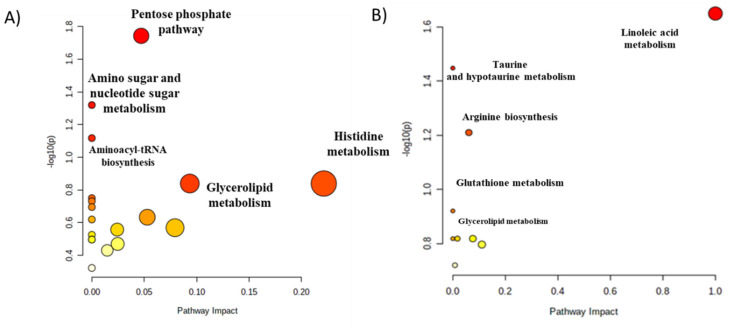
The results of biochemical pathways analysis performed for metabolites detected by GC–MS (**panel A**) and by LC-QTOF-MS (**panel B**).

Pathway mapping using MetaboAnalyst 5.0 showed that significant metabolites identified in the present study using GC–MS (Figure 2, panel A) and in the previous study [11] using LC-QTOF-MS (Figure 2, panel B) belong to different metabolic pathways. It proves that both analytical platforms provide complementary results. Pathway analysis based on GC–MS data revealed several pathways, including glycerolipid metabolism, histidine metabolism, pentose phosphate pathway or amino sugar and nucleotide sugar metabolism, where disturbance may lead to T2DM development. Metabolic pathways pointed based on LC–MS data are also important in the development of T2DM. Detailed information about all performed pathway analyses are provided in Figure 2, Table 4 and Table 5.

The graphics contain all the matched pathways arranged by log(*p*) values (from pathway enrichment analysis) on the y-axis, and pathway impact values (from pathway topology analysis) on the x-axis. The node colour is related to the pathway *p*-value, and the node radius is determined based on the pathway impact value. The dots symbolise modulated pathways (one dot—one biochemical pathway). Their localisation is dependent on the impact of a pathway in the whole analysis and its statistical significance. The pathway impact is the impact value calculated from pathway topology analysis. The total/maximum importance of each pathway is 1.

## 4. Discussion

The pathogenesis of T2DM is complex [2], and is characterised by the following triad: genetic predisposition, environmental factors and acquired organ dysfunction. The genetic predisposition to T2DM accompanied by such environmental factors as diet and sedentary lifestyle, along with β-cell dysfunction, IR and hepatic glucose production, leads to prediabetes and T2DM [1].

Meta-analyses of genome-wide association studies have confirmed that the rs340874 single-nucleotide polymorphism in the *PROX1* gene is associated with fasting glycemia and T2DM [26]. However, the mechanism of this link is not fully established [9]. Genetic predisposition–diet interaction is considered as one of the components of this mechanism [27]. We have previously investigated the effect of the meal-challenge test on the plasma metabolome of individuals with a different genetic predisposition to T2DM in the *PROX1* gene using LC-QTOF-MS [11]. However, to the best of our knowledge, how *PROX1* SNPs is related to the postprandial changes of plasma metabolites has not yet been investigated by GC–MS, an analytical platform complementary to LC–MS in metabolomics studies [28].

**Figure 3:**
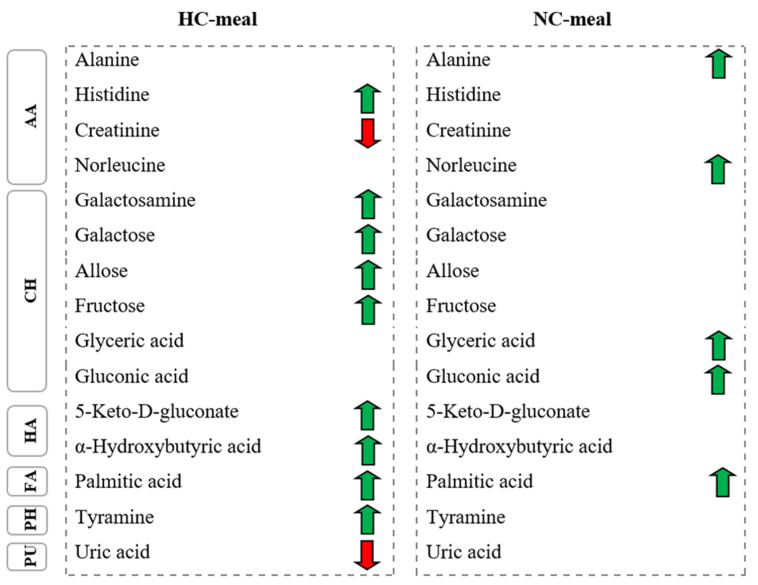
The summary of metabolic alternations observed after NC- and HC-meal intake. AA—Amino acids, CH—Carbohydrates, HA—Hydroxy acids, DI—Dicarboxylic acids, FA—Fatty acids, PH—Phenethylamines, PU—Purines, KA—Keto acids. Red arrows indicate a decrease, whereas green arrows indicate an increase in the metabolite level in HR genotype compared to the LR genotype. Transverse stripes show metabolites common between two meals.

Therefore, we selected this technique to measure plasma metabolites at fasting and postprandial state to explore the impact of the rs340874 SNPs in the *PROX1* gene on human metabolism. Metabolites detected in the present study were found to be associated with several metabolic pathways (Figure 2), which have already been linked to prediabetes and T2DM [16]. Furthermore, these significant metabolites belong to different chemical classes, i.e., amino acids, carbohydrates, hydroxy acids and others (Figure 3), and using such division are described below.

Carbohydrates are one of the three macronutrients in the human diet, along with protein and fat. As the role of dietary carbohydrates in the development and maintenance of T2DM receives now increasing attention [29], the participants of this study underwent an HC-meal challenge test. The progress of T2DM is often explained by excessive consumption of high-carbohydrates and high-calorie diets [30]. There is a lot of systematic reviews and meta-analyses describing the effect of low carbohydrate diet [29,30] in comparison to normal or high carbohydrate diet [11,27,31] in patients with T2DM, but only some of these studies described the post-meal changes in patients with a genetic predisposition to T2DM [1,11,27].

According to the literature review, among compounds involved in carbohydrate metabolism, the levels of malic acid, glucose, mannose, fructose, inositol, sorbitol, xylose, gluconic acid, glucuronic acid and fumaric acid were increased [32,33,34], whereas the levels of pyruvic acid, deoxygalactose, glycerol-3-phosphate and 1,5-anhydroglucitol were decreased in T2DM [28,32,35]. The levels of other metabolites such as citric acid, lactic acid, acetic acid and deoxyglucose were increased in some studies [36,37] and decreased in others [32,36]. Some of the metabolites mentioned above were also found significant in the presented study. After NC-meal intake, we observed changes in two metabolites involved in carbohydrates metabolism, whereas after HC-meal intake, we found significantly higher levels of four metabolites in the HR men (Table 2).

In the present study, 30, 60 and 120 min after HC-meal intake, the HR-genotype carriers presented significantly higher plasma levels for galactose, fructose, D-allose and galactosamine (GlycA). Galactose is a C-4 epimer of glucose and can be rapidly converted to glucose through the Leloir pathway [38]. Postprandially increased galactose metabolism may lead to a long-term, gradual increase in plasma glucose and may contribute to IR [39]. Human studies have shown that in addition to galactose, fructose may contribute negatively to blood glucose homeostasis by causing IR in the liver [40]. It was confirmed in another study that this six-carbon sugar, when ingested in a high amount, increases the risk of T2DM and other metabolic diseases [41]. Fructose, once ingested, can be oxidised, converted to glucose or lactic acid or enter de novo lipogenesis. These metabolic pathways could lead to the development of metabolic disorders. After ingestion of large amounts of fructose, an increased level of hepatic acetyl CoA leads to increased production of very-low-density lipoprotein and triglycerides, associated with T2DM. GlycA can be a predictor of the future development of T2DM [42], but conclusive data on the relation of GlycA with IR or insulin secretion are missing [43]. Connelly et al. [44] also concluded that further research is needed to understand the inflammatory pathophysiology of T2DM and the ability of GlycA to improve prediction models for T2DM. Low-grade inflammation is known to trigger the development of IR and loss of β-cell function, and both are proposed to be involved in the pathogenesis of T2DM (reviewed here [45,46]).

A recent study shows that fructose-mediated generation of uric acid causes mitochondrial oxidative stress that stimulates fat accumulation independent of excessive caloric intake [40]. Research into the relationship between uric acid and T2DM is the most controversial. A lot of studies confirmed that uric acid was related to an increased risk of T2DM [47,48]. On the other hand, in the study of Li et al. [49], who followed up 4412 nondiabetic patients for around 5 years to study serum urate changes in glucose metabolism, high concentrations of uric acid were not found to be related to an increased risk of T2DM. As shown in Figure 3, we observed a significantly lower uric acid level after HC-meal intake in HR-men.

The metabolism of carbohydrates and fats are closely connected. Plasma FFAs can be synthesised endogenously from excess carbohydrates in the process of de novo lipogenesis. This process is upregulated by insulin in the blood and downregulated by high levels of such hormones as adrenaline and glucagon. Fatty acids involved in this pathway have been recently characterised as a cause [50,51] and a consequence [52] of IR and T2DM. Moreover, it is well known that T2DM and lipid disorders (involving FFAs [53]) are closely associated.

However, different kinds of FFAs have different or even opposite effects on IR and T2DM. For example, saturated fatty acids (SFA) worsen insulin sensitivity and increase the risk of T2DM, but polyunsaturated fatty acids (PUFA), particularly n-3 fatty acids, improve IR [2] and are potentially protective against T2DM [54]. It should be kept in mind that plasma FFAs concentrations reflect their intake and balance between de novo FFA synthesis, storage as TAGs and their lipolysis. During digestion, TAGs are hydrolysed into mono- and diglycerides and FFAs [2].

In the study of Koska et al. [55], it was confirmed that high caloric diets enriched with saturated fatty acids (SFA) or carbohydrates induced whole-body IR in both normal and impaired glucose tolerant subjects. The metabolic response to SFAs can be associated with the induction of serine-phosphorylation through activation of specific serine kinases, resulting in decreased activity of insulin-regulated glucose transporter-4 (GLUT-4) and consequent lower glucose uptake [56]. SFAs can also affect insulin sensitivity by altering the membrane lipid composition, leading to the disorientation of membrane glucose transporter molecules [57].

In our study, HR genotype carriers presented a significantly higher level of palmitic acid (PA) 60 min after both meals. Literature data indicate that impaired insulin secretion, impaired insulin sensitivity and glucose intolerance are strongly associated with elevated plasma levels of saturated FFAs (including palmitic and stearic acid) [3,5,58]. The elevated level of plasma PA may contribute to hyperinsulinemia and consequently the development of IR. It has also been reported that IR could be induced by the increase of circulating FFAs, which inhibit glucose transport and phosphorylation activity and down-regulate glycogen synthesis and glucose oxidation in muscle [59]. In the prospective multi-ethnic cohort study [60], the associations of metabolic perturbations in fatty acid metabolism with a 5-year risk of incident type 2 diabetes, before and after adjusting for insulin sensitivity and IR, were investigated. The researchers found out that PA had the strongest association with the risk of T2DM. Our results also support this observation, as plasma PA level was found to be postprandially increased in HR gene risk carriers up to 5 years prior to T2DM onset. Excessive fatty acid oxidation elevates the intramitochondrial acetyl CoA/CoA and NADH/NAD^+^ ratios and inhibits pyruvate oxidation [61]. Thus, lactate accumulation may occur. This inefficient fat oxidation may cause an increased level of alpha-hydroxybutyric acid (α-HB). This metabolite is a byproduct of α ketobutyric acid synthesis, a product of amino acid catabolism (threonine and methionine) and glutathione anabolism (cysteine formation pathway) in hepatic tissue. α-HB was previously identified as a marker [16,62,63] and predictor [64,65] of T2DM. In the present study, we also observed a significantly higher level of this metabolite in HR-genotype carriers 60 and 120 min after HC-meal. Consequently, an accurate determination of PA and α-HB can be essential for the early diagnosis of T2DM development, which is an important finding of this study.

Amino acids (AAs) are crucial biological compounds that play a key metabolic and physiological role in all living organisms [66]. It was confirmed that these metabolites are useful diagnostic markers because they are considerably altered in prediabetes state and continue to increase during T2DM progression [67]. The Framingham Offspring Study has shown that elevated AAs were able to predict an increased risk of T2DM up to 12 years prior to disease onset [68]. A less consistent finding is the association of prediabetes or T2DM with higher levels of lysine [69,70,71], histidine [69] or proline [14,34,70,72].

Our study supported the observations of the relationship between amino acid levels and the risk of T2DM. We noticed higher plasma level of alanine (Ala) and norleucine in HR-genotype carriers after NC-meal. Additionally, we have observed an increased level of histidine in HR-genotype carriers after HC-meal. As mentioned above, accumulation of these metabolites after meal intake may lead to activation of pathways involved in T2DM development.

Alanine is a proteinogenic amino acid which level was found elevated in HR-men in 60 and 120 min after NC-meal intake. It is synthesised from pyruvate and amino acids (mainly BCAAs) primarily in skeletal muscle and intestines and is used for gluconeogenesis in the liver [73]. Therefore, postprandially increased level of Ala in plasma may enhance gluconeogenesis and contribute to fasting hyperglycemia development.

After HC-meal intake, the HR-genotype carriers presented a significantly higher plasma level of histidine and a lower postprandial level of creatinine. Although plasma histidine is an appetite-controlling factor, it also provokes a brain signal to the liver that decreases the expression of gluconeogenic enzymes—most notably glucose-6-phosphatase–and thereby reduces hepatic glucose output. On the other hand, a small Japanese prospective study reached the conclusion that higher dietary intake of BCAAs was a predictor of a lower risk of T2DM. Therefore, arguably, the true dietary determinant of risk might be the histidine ratio to BCAAs or total neutral amino acids. In the diet or in plasma, such a ratio might predict risk better than either histidine or BCAAs per se. On the other hand, studies describe the positive of histidine in preventing T2DM [74].

Many published articles confirm our results for creatinine. For example, in 2009, Harita et al. [75] confirmed that low serum creatinine levels were associated with an increased risk of type 2 diabetes mellitus and dysglycemia. This was also confirmed in the recent studies conducted by Takeuchi et al. [76] and Chutani and Pande [77]. Creatinine is produced after the pyrophosphate cleavage of phosphocreatine to produce energy for muscle activity. Therefore, a lower serum creatinine level might reflect a lower volume of skeletal muscle which means fewer target sites for insulin. In addition, it may partially explain the pathogenesis of T2DM associated with lower serum creatinine. Consequently, an accurate determination of these metabolites can be essential for the early diagnosis of prediabetes or T2DM.

Among other significant metabolites, we observed elevated tyramine level 30 and 60 min after HC-meal in HR-men. This compound is derived from tyrosine and has a good effect on human health. Intake of tyramine might bring a benefit, especially when glucotoxicity or lipotoxicity need to be reduced, i.e., in diabetic and obese condition [78]. In 2003 Visentin et al. [79] reported that tyramine stimulates in vitro glucose transport in adipocytes, cardiomyocytes and skeletal muscle and improves in vivo glucose utilisation in rats. Carpene et al. [78] observed that after 12 weeks of tyramine supplementation, non-fasting blood glucose was decreased, but the supplementation did not modify glucose tolerance or fasting glucose level, insulin or circulating lipids.

Our study had several strengths and limitations. One of the strengths was the use of a well-established GC–MS platform, which allowed us to discover novel metabolites, previously not detected using LC-QTOF-MS. A major limitation of our study is a relatively small sample size, but as it was mentioned in our previous studies performed on the same group of patients, it is difficult to find healthy risk genotypes carriers since the general CC genotype frequency is around 6% [9,11]. Furthermore, due to the long and laborious protocols of the meal challenge test, it is also challenging to recruit volunteers willing to participate in this type of study.

## 5. Conclusions and Future Prospects

Plasma GC–MS profiling provided an efficient way of depicting metabolic perturbations observed in HR-genotype carriers after meals intake. Considering the fact that several of the studied HR-genotype carriers became prediabetic within 5 years, the presented results are of high importance. Alterations in the level of several metabolites can be an early metabolic disturbance predicting early stages of T2DM development, and even some of them, such as α-HB, gluconic acid and PA, can be early predictors of the *PROX1*-related risk of T2DM in healthy people. The obtained result may also help to establish diet recommendations for individuals carrying the T2DM-risk allele in the *PROX1* gene. However, further investigations are required to design an optimal diet. To evaluate the clinical utility of altered metabolites as potential markers of genetic predisposition to T2DM, a targeted method for the determination of significant metabolites should be developed and applied in a large cohort of patients to measure fasting and postprandial plasma samples.

## Figures and Tables

**Table 1:** Baseline characteristics of the study groups.

	LR Genotype Carriers	HR Genotype Carriers	*p*
Age (years)	35.8 ± 6.9	35.2 ± 9.0	0.88
Weight (kg)	91.8 ± 22.2	93.6 ± 23.5	0.89
Body mass index (BMI) (kg/m2)	28.1 ± 5.4	29.1 ± 7.8	0.79
Body fat content (%)	23.6 ± 7.7	23.8 ± 9.6	0.96
Fat-free mass (%)	76.29 ± 10.1	74.3 ± 9.0	0.88
Waist (cm)	105.5 ± 21.5	107.4 ± 21.3	0.94
Hip (cm)	108.0 ± 10.0	104.4 ± 15.6	0.79
WHR	0.97 ± 0.11	1.03 ± 0.16	0.66
Fasting glucose concentration (mg/dl)	84.7 ± 5.1	86.2 ± 7.6	0.65
Fasting insulin activity (IU/mL)	9.7 ± 7.5	10.4± 8.7	0.87
HOMA-IR	2.1 ± 1.8	2.2 ± 1.9	0.89
HOMA-B	150.2 ± 81.4	188.2 ± 156.3	0.53
HbA1c	5.2 ± 0.3	5.2 ± 0.5	0.77

**Table 2:** The percentage differences in postprandial plasma metabolite levels after HC-meal intake in the *PROX1* low- (LR) and high-risk (HR) genotype men. Metabolites that showed statistical significance after Wilcoxon signed-rank test. Mass found in the Human Metabolome Database (HMDB) (http://www.hmdb.caaccessed, on 20 April 2021); RT, retention time expressed in minutes; *p* value; * *p* value for between-groups comparison (Mann–Whitney U test); CV, coefficient of variation of the metabolites in the QC samples; FC, fold change in the comparison. Eight individuals from the HR group participated in HC-meal.

Compound	Mass (DB)	Molecular Formula	RT (min)	Time after Meal	HR-HC-Meal		LR-HC-Meal		CV (%)	* *p* Value	HMDB Code
					*p* Value	FC	*p* Value	FC			
Amino acids, peptides and analogues											
Histidine	155.1546	C_6_H_9_N_3_O_2_	17.20	0′–60′	0.04	1.90	0.19	1.52	27	0.02	HMDB00177
				0′–120′	0.02	2.20	0.19	1.30		1.00	
Creatinine	113.1179	C_4_H_7_N_3_O	13.60	0′–30′	0.04	0.70	0.63	0.89	22	0.43	HMDB00562
Carbohydrates and carbohydrate conjugates											
Galactosamine	221.2078	C_8_H_15_NO_6_	17.40	0′–30′	0.008	1.61	0.13	1.50	9	0.66	HMDB00803
				0′–60′	0.008	1.91	0.19	1.49		0.54	
				0′–120′	0.008	1.89	0.31	1.27		0.43	
Galactose	180.1559	C_6_H_12_O_6_	17.30	0′–30′	0.008	1.61	0.19	1.45	10	0.54	HMDB00143
				0′–60′	0.008	1.89	0.31	1.43		0.66	
				0′–120′	0.008	1.95	0.44	1.22		0.43	
Allose	180.1559	C_6_H_12_O_6_	17.54	0′–30′	0.008	2.89	0.44	1.46	9	0.66	HMDB01151
				0′–60′	0.008	2.04	0.44	1.50		0.54	
				0′–120′	0.008	1.92	0.63	0.53			
Fructose	180.1559	C_6_H_12_O_6_	17.00	0′–30′	0.008	1.82	0.63	1.29	13	0.13	HMDB00660
				0′–60′	0.02	1.60	0.44	1.44		0.33	
				0′–120′	0.008	1.78	0.86	1.40		0.54	
Hydroxy acids and derivative											
5-Keto-gluconate	194.1394	C_6_H_10_O_7_	17.53	0′–30′	0.008	1.66	0.13	1.51	9	0.43	HMDB11731
				0′–60′	0.008	2.02	0.31	1.49		0.54	
				0′–120′	0.008	1.92	0.31	1.28		0.43	
α-Hydroxybutyric acid	104.1045	C_4_H_8_O_3_	7.70	0′–60′	0.008	1.21	0.44	1.15	10	0.66	HMDB00008
				0′–120′	0.008	1.33	0.81	1.05		0.54	
Fatty acids and conjugates											
Palmitic acid	256.424	C_16_H_32_O_2_	18.85	0′–60′	0.02	1.42	0.19	0.39	22	0.54	HMDB00220
Phenethylamines											
Tyramine	137.179	C_8_H_11_NO	17.60	0′–30′	0.008	1.66	0.81	1.03	11	0.03	HMDB00306
				0′–60′	0.008	2.97	0.81	1.02		0.05	
				0′–120′	0.008	1.95	1.00	0.87		0.08	
Purines and purine derivatives											
Uric acid	168.1103	C_5_H_4_N_4_O_3_	19.30	0′–120′	0.04	0.53	0.58	1.25	27	0.54	HMDB00289

**Table 3:** The percentage differences in postprandial plasma metabolite levels after NC-meal intake in the *PROX1* low- (LR) and high-risk (HR) genotype men. Metabolites that showed statistical significance after Wilcoxon signed-rank test. Mass found in the Human Metabolome Database (HMDB) (http://www.hmdb.ca/accessed, on 20 April 2021); RT, retention time expressed in minutes; *p* value; * *p* value for between-groups comparison (Mann–Whitney U test); CV, coefficient of variation of the metabolites in the QC samples; FC, fold change in the comparison. Ten individuals from the HR group participated in NC-meal (six common with HC-meal).

Compound	Mass (DB)	Molecular Formula	RT (min)	Time after Meal	HR-NC-Meal		LR-NC-Meal		CV (%)	* *p* Value Groups	HMDB Code
					*p* Value	FC	*p* Value	FC			
Amino acids, peptides and analogues											
Alanine	146.1876	C_6_H_14_N_2_O_2_	7.4	00′–;-600′–;	0.04	2.00	0.13	1.26	25	0.91	HMDB00161
				00′–;-1200′–;	0.02	3.70	0.88	1.21		0.01	
Norleucine	131.173	C_6_H_13_NO_2_	8.53	00′–;-300′–;	0.006	2.30	0.88	0.95	24	0.11	HMDB01645
				00′–;-600′–;	0.04	2.27	0.25	1.75		0.60	
Carbohydrates and carbohydrate conjugates											
Gluconic acid	196.1553	C_6_H_12_O_7_	17.62	00′–;-600′–;	0.04	1.88	0.63	1.17	14	0.11	HMDB00625
				00′–;-1200′–;	0.01	3.47	0.13	2.42		0.17	
Glyceric acid	106.0773	C_3_H_6_O_4_	9.80	00′–;-1200′–;	0.002	1.77	0.13	1.73	10	0.02	HMDB00139
Fatty acids and conjugates											
Palmitic acid	256.424	C_16_H_32_O_2_	18.85	00′–;-600′–;	0.01	1.62	0.13	0.62	29	0.91	HMDB00220

**Table 4:** Metabolic pathways corresponding to significant metabolites identified in plasma samples with GC–MS.

Pathway Name	No. of Metabolites in the Pathway	No. of Metabolites Detected in Plasma	*p*-Value	Pathway Impact
Pentose phosphate pathway	22	2	0.018	0.047
Amino sugar and nucleotide sugar metabolism	37	2	0.048	0.000
Aminoacyl-tRNA biosynthesis	48	2	>0.05	0.000
Glycerolipid metabolism	16	1	>0.05	0.093
Histidine metabolism	16	1	>0.05	0.221
Selenocompound metabolism	20	1	>0.05	0.000
beta-Alanine metabolism	21	1	>0.05	0.000
Propanoate metabolism	23	1	>0.05	0.000
Galactose metabolism	27	1	>0.05	0.053
Alanine, aspartate and glutamate metabolism	28	1	>0.05	0.000
Glyoxylate and dicarboxylate metabolism	32	1	>0.05	0.080
Glycine, serine and threonine metabolism	33	1	>0.05	0.024
Biosynthesis of unsaturated fatty acids	36	1	>0.05	0.000
Fatty acid elongation	39	1	>0.05	0.000
Fatty acid degradation	39	1	>0.05	0.000
Tyrosine metabolism	42	1	>0.05	0.024
Fatty acid biosynthesis	47	1	>0.05	0.014
Purine metabolism	65	1	>0.05	0.000

**Table 5:** Metabolic pathways corresponding to significant metabolites identified in plasma samples with LC–MS.

Pathway Name	No. of Metabolites in the Pathway	No. of Metabolites Detected in the Plasma	*p*-Value	Pathway Impact
Linoleic acid metabolism	5	1	0.022	1.000
Taurine and hypotaurine metabolism	8	1	0.036	0
Arginine biosynthesis	14	1	0.062	0.061
Glutathione metabolism	28	1	0.120	0
Biosynthesis of unsaturated fatty acids	36	1	0.152	0
Glycerophospholipid metabolism	36	1	0.152	0.017
Arachidonic acid metabolism	36	1	0.152	0.076
Arginine and proline metabolism	38	1	0.160	0.111
Primary bile acid biosynthesis	46	1	0.190	0.008

## Data Availability

The datasets analyzed during the current study are available from the corresponding author on reasonable request.

## References

[1] Ericson U., Hindy G., Drake I., Schulz C.-A., Brunkwall L., Hellstrand S., Almgren P., Orho-Melander M. (2018). Dietary and genetic risk scores and incidence of type 2 diabetes. Genes Nutr..

[2] Sobczak A., A Blindauer C., J Stewart A. (2019). Changes in Plasma Free Fatty Acids Associated with Type-2 Diabetes. Nutrients.

[3] Castro-Correia C., Sousa S., Norberto S., Matos C., Domingues V.F., Fontoura M., Calhau C. (2017). The Fatty Acid Profile in Patients with Newly Diagnosed Diabetes: Why It Could Be Unsuspected. Int. J. Pediatrics.

[4] Yang S.J., Kwak S.-Y., Jo G., Song T.-J., Shin M.-J. (2018). Serum metabolite profile associated with incident type 2 diabetes in Koreans: Findings from the Korean Genome and Epidemiology Study. Sci. Rep..

[5] Lu Y., Wang Y., Ong C.-N., Subramaniam T., Choi H.W., Yuan J.-M., Koh W.-P., Pan A. (2016). Metabolic signatures and risk of type 2 diabetes in a Chinese population: An untargeted metabolomics study using both LC-MS and GC-MS. Diabetologia.

[6] Dietrich S., Jacobs S., Zheng J.-S., Meidtner K., Schwingshackl L., Schulze M.B. (2019). Gene-lifestyle interaction on risk of type 2 diabetes: A systematic review. Obes. Rev..

[7] Goto T., Elbahrawy A., Furuyama K., Horiguchi M., Hosokawa S., Aoyama Y., Tsuboi K., Sakikubo M., Hirata K., Masui T. (2017). Liver-specific PROX1 inactivation causes hepatic injury and glucose intolerance in mice. FEBS Lett..

[8] Harvey N.L., Srinivasan R.S., Dillard M.E., Johnson N.C., Witte M.H., Boyd K., Sleeman M.W., Oliver G. (2005). Lymphatic vascular defects promoted by *PROX1*1 haploinsufficiency cause adult-onset obesity. Nat. Genet..

[9] Kretowski A., Adamska E., Maliszewska K., Wawrusiewicz-Kurylonek N., Citko A., Goscik J., Bauer W., Wilk J., Golonko A., Waszczeniuk M. (2015). The rs340874 *PROX1* type 2 diabetes mellitus risk variant is associated with visceral fat accumulation and alterations in postprandial glucose and lipid metabolism. Genes Nutr..

[10] Hindy G., Sonestedt E., Ericson U., Jing X.J., Zhou Y., Hansson O., Renström E., Wirfält E., Orho-Melander M. (2012). Role of TCF7L2 risk variant and dietary fibre intake on incident type 2 diabetes. Diabetologia.

[11] Adamska-Patruno E., Godzien J., Ciborowski M., Samczuk P., Bauer W., Siewko K., Gorska M., Barbas C., Kretowski A. (2019). The Type 2 Diabetes Susceptibility *PROX1* Gene Variants Are Associated with Postprandial Plasma Metabolites Profile in Non-Diabetic Men. Nutrients.

[12] Long J., Yang Z., Wang L., Han Y., Peng C., Yan C., Yan D. (2020). Metabolite biomarkers of type 2 diabetes mellitus and pre-diabetes: A systematic review and meta-analysis. BMC Endocr. Disord..

[13] Haeusler R.A., Astiarraga B., Camastra S., Accili D., Ferrannini E. (2013). Human insulin resistance is associated with increased plasma levels of 12α-hydroxylated bile acids. Diabetes.

[14] Tai E.S., Tan M.L., Stevens R.D., Low Y.L., Muehlbauer M.J., Goh D.L., Ilkayeva O.R., Wenner B.R., Bain J.R., Lee J.J. (2010). Insulin resistance is associated with a metabolic profile of altered protein metabolism in Chinese and Asian-Indian men. Diabetologia.

[15] Gar C., Rottenkolber M., Prehn C., Adamski J., Seissler J., Lechner A. (2018). Serum and plasma amino acids as markers of prediabetes, insulin resistance, and incident diabetes. Crit. Rev. Clin. Lab. Sci..

[16] Dorcely B., Katz K., Jagannathan R., Chiang S.S., Oluwadare B., Goldberg I.J., Bergman M. (2017). Novel biomarkers for prediabetes, diabetes, and associated complications. Diabetes Metab. Syndr. Obes. Targets Ther..

[17] Mook-Kanamori D.O., de Mutsert R., Rensen P.C.N., Prehn C., Adamski J., den Heijer M., le Cessie S., Suhre K., Rosendaal F.R., van Dijk K.W. (2016). Type 2 diabetes is associated with postprandial amino acid measures. Arch. Biochem. Biophys..

[18] Wang H., Zhang H., Yao L., Cui L., Zhang L., Gao B., Liu W., Wu D., Chen M., Li X. (2018). Serum metabolic profiling of type 2 diabetes mellitus in Chinese adults using an untargeted GC/TOFMS. Clin. Chim. Acta.

[19] Kvitvang H.F., Kristiansen K.A., Lien S.K., Bruheim P. (2014). Quantitative analysis of amino and organic acids by methyl chloroformate derivatization and GC-MS/MS analysis. Methods Mol. Biol..

[20] Hoving L.R., Heijink M., van Harmelen V., van Dijk K.W., Giera M. (2018). GC-MS Analysis of Short-Chain Fatty Acids in Feces, Cecum Content, and Blood Samples. Methods Mol. Biol..

[21] Ciborowski M., Adamska E., Rusak M., Godzien J., Wilk J., Citko A., Bauer W., Gorska M., Kretowski A. (2015). CE-MS-based serum fingerprinting to track evolution of type 2 diabetes mellitus. Electrophoresis.

[22] Sidorkiewicz I., Niemira M., Maliszewska K., Erol A., Bielska A., Szalkowska A., Adamska-Patruno E., Szczerbinski L., Gorska M., Kretowski A. (2020). Circulating miRNAs as a Predictive Biomarker of the Progression from Prediabetes to Diabetes: Outcomes of a 5-Year Prospective Observational Study. J. Clin. Med..

[23] Lu J., Varghese R.T., Zhou L., Vella A., Jensen M.D. (2017). Glucose tolerance and free fatty acid metabolism in adults with variations in TCF7L2 rs7903146. Metabolism.

[24] Hagströmer M., Oja P., Sjöström M. (2006). The International Physical Activity Questionnaire (IPAQ): A study of concurrent and construct validity. Public Health Nutr..

[25] Dudzik D., Iglesias Platas I., Izquierdo Renau M., Balcells Esponera C., Del Rey Hurtado de Mendoza B., Lerin C., Ramón-Krauel M., Barbas C. (2020). Plasma Metabolome Alterations Associated with Extrauterine Growth Restriction. Nutrients.

[26] Mahajan A., Go M.J., Zhang W., Below J.E., Gaulton K.J., Ferreira T., Horikoshi M., Johnson A.D., Ng M.C., Prokopenko I. (2014). Genome-wide trans-ancestry meta-analysis provides insight into the genetic architecture of type 2 diabetes susceptibility. Nat. Genet..

[27] Adamska-Patruno E., Samczuk P., Ciborowski M., Godzien J., Pietrowska K., Bauer W., Gorska M., Barbas C., Kretowski A. (2019). Metabolomics Reveal Altered Postprandial Lipid Metabolism After a High-Carbohydrate Meal in Men at High Genetic Risk of Diabetes. J. Nutr..

[28] Fiehn O. (2016). Metabolomics by Gas Chromatography-Mass Spectrometry: Combined Targeted and Untargeted Profiling. Curr. Protoc. Mol. Biol..

[29] Czyżewska-Majchrzak Ł., Grzelak T., Kramkowska M., Czyżewska K., Witmanowski H. (2014). The use of low-carbohydrate diet in type 2 diabetes—Benefits and risks. Ann. Agric. Environ. Med..

[30] Goldenberg J.Z., Day A., Brinkworth G.D., Sato J., Yamada S., Jönsson T., Beardsley J., Johnson J.A., Thabane L., Johnston B.C. (2021). Efficacy and safety of low and very low carbohydrate diets for type 2 diabetes remission: Systematic review and meta-analysis of published and unpublished randomized trial data. BMJ.

[31] Tay J., Luscombe-Marsh N.D., Thompson C.H., Noakes M., Buckley J.D., Wittert G.A., Yancy W.S., Brinkworth G.D. (2015). Comparison of low- and high-carbohydrate diets for type 2 diabetes management: A randomized trial. Am. J. Clin. Nutr..

[32] Drogan D., Dunn W.B., Lin W., Buijsse B., Schulze M.B., Langenberg C., Brown M., Floegel A., Dietrich S., Rolandsson O. (2015). Untargeted metabolic profiling identifies altered serum metabolites of type 2 diabetes mellitus in a prospective, nested case control study. Clin. Chem..

[33] Suhre K., Meisinger C., Döring A., Altmaier E., Belcredi P., Gieger C., Chang D., Milburn M.V., Gall W.E., Weinberger K.M. (2010). Metabolic footprint of diabetes: A multiplatform metabolomics study in an epidemiological setting. PLoS ONE.

[34] Xu F., Tavintharan S., Sum C.F., Woon K., Lim S.C., Ong C.N. (2013). Metabolic signature shift in type 2 diabetes mellitus revealed by mass spectrometry-based metabolomics. J. Clin. Endocrinol. Metab..

[35] Fiehn O., Garvey W.T., Newman J.W., Lok K.H., Hoppel C.L., Adams S.H. (2010). Plasma metabolomic profiles reflective of glucose homeostasis in non-diabetic and type 2 diabetic obese African-American women. PLoS ONE.

[36] Diao C., Zhao L., Guan M., Zheng Y., Chen M., Yang Y., Lin L., Chen W., Gao H. (2014). Systemic and characteristic metabolites in the serum of streptozotocin-induced diabetic rats at different stages as revealed by a (1)H-NMR based metabonomic approach. Mol. Biosyst..

[37] Gogna N., Krishna M., Oommen A.M., Dorai K. (2015). Investigating correlations in the altered metabolic profiles of obese and diabetic subjects in a South Indian Asian population using an NMR-based metabolomic approach. Mol. Biosyst..

[38] Holden H.M., Rayment I., Thoden J.B. (2003). Structure and function of enzymes of the Leloir pathway for galactose metabolism. J. Biol. Chem..

[39] Blaak E.E., Antoine J.M., Benton D., Björck I., Bozzetto L., Brouns F., Diamant M., Dye L., Hulshof T., Holst J.J. (2012). Impact of postprandial glycaemia on health and prevention of disease. Obes. Rev. Off. J. Int. Assoc. Study Obes..

[40] Johnson R.J., Nakagawa T., Sanchez-Lozada L.G., Shafiu M., Sundaram S., Le M., Ishimoto T., Sautin Y.Y., Lanaspa M.A. (2013). Sugar, uric acid, and the etiology of diabetes and obesity. Diabetes.

[41] Ang B., Yu G. (2017). The Role of Fructose in Type 2 Diabetes and Other Metabolic DIseases. Nutr. Food Sci..

[42] Akinkuolie A.O., Pradhan A.D., Buring J.E., Ridker P.M., Mora S. (2015). Novel protein glycan side-chain biomarker and risk of incident type 2 diabetes mellitus. Arter. Thromb. Vasc. Biol..

[43] Lorenzo C., Festa A., Hanley A.J., Rewers M.J., Escalante A., Haffner S.M. (2017). Novel Protein Glycan-Derived Markers of Systemic Inflammation and C-Reactive Protein in Relation to Glycemia, Insulin Resistance, and Insulin Secretion. Diabetes Care.

[44] Connelly M.A., Gruppen E.G., Wolak-Dinsmore J., Matyus S.P., Riphagen I.J., Shalaurova I., Bakker S.J., Otvos J.D., Dullaart R.P. (2016). GlycA, a marker of acute phase glycoproteins, and the risk of incident type 2 diabetes mellitus: PREVEND study. Clin. Chim. Acta.

[45] Hameed I., Masoodi S.R., Mir S.A., Nabi M., Ghazanfar K., Ganai B.A. (2015). Type 2 diabetes mellitus: From a metabolic disorder to an inflammatory condition. World J. Diabetes.

[46] Lontchi-Yimagou E., Sobngwi E., Matsha T.E., Kengne A.P. (2013). Diabetes mellitus and inflammation. Curr. Diabetes Rep..

[47] Bombelli M., Quarti-Trevano F., Tadic M., Facchetti R., Cuspidi C., Mancia G., Grassi G. (2018). Uric acid and risk of new-onset metabolic syndrome, impaired fasting glucose and diabetes mellitus in a general Italian population: Data from the Pressioni Arteriose Monitorate E Loro Associazioni study. J. Hypertens..

[48] Anothaisintawee T., Lertrattananon D., Thamakaison S., Reutrakul S., Ongphiphadhanakul B., Thakkinstian A. (2017). Direct and Indirect Effects of Serum Uric Acid on Blood Sugar Levels in Patients with Prediabetes: A Mediation Analysis. J. Diabetes Res..

[49] Li X., Meng X., Gao X., Pang X., Wang Y., Wu X., Deng X., Zhang Q., Sun C., Li Y. (2018). Elevated Serum Xanthine Oxidase Activity Is Associated With the Development of Type 2 Diabetes: A Prospective Cohort Study. Diabetes Care.

[50] Wilding J.P. (2007). The importance of free fatty acids in the development of Type 2 diabetes. Diabet. Med..

[51] Boden G. (2011). Obesity, insulin resistance and free fatty acids. Curr. Opin. Endocrinol. Diabetes Obes..

[52] Carmena R. (2005). Type 2 diabetes, dyslipidemia, and vascular risk: Rationale and evidence for correcting the lipid imbalance. Am. Heart J..

[53] Wyne K.L. (2003). Free fatty acids and type 2 diabetes mellitus. Am. J. Med..

[54] Djoussé L., Biggs M.L., Lemaitre R.N., King I.B., Song X., Ix J.H., Mukamal K.J., Siscovick D.S., Mozaffarian D. (2011). Plasma omega-3 fatty acids and incident diabetes in older adults. Am. J. Clin. Nutr..

[55] Koska J., Ozias M.K., Deer J., Kurtz J., Salbe A.D., Harman S.M., Reaven P.D. (2016). A human model of dietary saturated fatty acid induced insulin resistance. Metabolism.

[56] Kennedy A., Martinez K., Chuang C.-C., LaPoint K., McIntosh M. (2008). Saturated Fatty Acid-Mediated Inflammation and Insulin Resistance in Adipose Tissue: Mechanisms of Action and Implications. J. Nutr..

[57] van der Kolk B.W., Goossens G.H., Jocken J.W., Blaak E.E. (2016). Altered skeletal muscle fatty acid handling is associated with the degree of insulin resistance in overweight and obese humans. Diabetologia.

[58] Gustavo Vazquez-Jimenez J., Chavez-Reyes J., Romero-Garcia T., Zarain-Herzberg A., Valdes-Flores J., Manuel Galindo-Rosales J., Rueda A., Guerrero-Hernandez A., Alberto Olivares-Reyes J. (2016). Palmitic acid but not palmitoleic acid induces insulin resistance in a human endothelial cell line by decreasing SERCA pump expression. Cell. Signal..

[59] Forouhi N.G., Imamura F., Sharp S.J., Koulman A., Schulze M.B., Zheng J., Ye Z., Sluijs I., Guevara M., Huerta J.M. (2016). Association of Plasma Phospholipid n-3 and n-6 Polyunsaturated Fatty Acids with Type 2 Diabetes: The EPIC-InterAct Case-Cohort Study. PLoS Med..

[60] Qureshi W., Santaren I.D., Hanley A.J., Watkins S.M., Lorenzo C., Wagenknecht L.E. (2019). Risk of diabetes associated with fatty acids in the de novo lipogenesis pathway is independent of insulin sensitivity and response: The Insulin Resistance Atherosclerosis Study (IRAS). BMJ Open Diabetes Res. Care.

[61] Fillmore N., Mori J., Lopaschuk G.D. (2014). Mitochondrial fatty acid oxidation alterations in heart failure, ischaemic heart disease and diabetic cardiomyopathy. Br. J. Pharmacol..

[62] Gall W.E., Beebe K., Lawton K.A., Adam K.-P., Mitchell M.W., Nakhle P.J., Ryals J.A., Milburn M.V., Nannipieri M., Camastra S. (2010). alpha-hydroxybutyrate is an early biomarker of insulin resistance and glucose intolerance in a nondiabetic population. PLoS ONE.

[63] Cobb J., Eckhart A., Motsinger-Reif A., Carr B., Groop L., Ferrannini E. (2016). α-Hydroxybutyric Acid Is a Selective Metabolite Biomarker of Impaired Glucose Tolerance. Diabetes Care.

[64] Varvel S.A., Pottala J.V., Thiselton D.L., Caffrey R., Dall T., Sasinowski M., McConnell J.P., Warnick G.R., Voros S., Graham T.E. (2014). Serum α-hydroxybutyrate (α-HB) predicts elevated 1 h glucose levels and early-phase β-cell dysfunction during OGTT. BMJ Open Diabetes Res. Care.

[65] Ferrannini E., Natali A., Camastra S., Nannipieri M., Mari A., Adam K.P., Milburn M.V., Kastenmüller G., Adamski J., Tuomi T. (2013). Early metabolic markers of the development of dysglycemia and type 2 diabetes and their physiological significance. Diabetes.

[66] Zhang S., Zeng X., Ren M., Mao X., Qiao S. (2017). Novel metabolic and physiological functions of branched chain amino acids: A review. J. Anim. Sci. Biotechnol..

[67] Fikri A.M., Smyth R., Kumar V., Al-Abadla Z., Abusnana S., Munday M.R. (2020). Pre-diagnostic biomarkers of type 2 diabetes identified in the UAE’s obese national population using targeted metabolomics. Sci. Rep..

[68] Wang T.J., Larson M.G., Vasan R.S., Cheng S., Rhee E.P., McCabe E., Lewis G.D., Fox C.S., Jacques P.F., Fernandez C. (2011). Metabolite profiles and the risk of developing diabetes. Nat. Med..

[69] Zhang X., Wang Y., Hao F., Zhou X., Han X., Tang H., Ji L. (2009). Human Serum Metabonomic Analysis Reveals Progression Axes for Glucose Intolerance and Insulin Resistance Statuses. J. Proteome Res..

[70] Zhou Y., Qiu L., Xiao Q., Wang Y., Meng X., Xu R., Wang S., Na R. (2013). Obesity and diabetes related plasma amino acid alterations. Clin. Biochem..

[71] Thalacker-Mercer A.E., Ingram K.H., Guo F., Ilkayeva O., Newgard C.B., Garvey W.T. (2014). BMI, RQ, diabetes, and sex affect the relationships between amino acids and clamp measures of insulin action in humans. Diabetes.

[72] Badoud F., Lam K.P., Perreault M., Zulyniak M.A., Britz-McKibbin P., Mutch D.M. (2015). Metabolomics Reveals Metabolically Healthy and Unhealthy Obese Individuals Differ in their Response to a Caloric Challenge. PLoS ONE.

[73] Holeček M. (2018). Branched-chain amino acids in health and disease: Metabolism, alterations in blood plasma, and as supplements. Nutr. Metab..

[74] Feng R.N., Niu Y.C., Sun X.W., Li Q., Zhao C., Wang C., Guo F.C., Sun C.H., Li Y. (2013). Histidine supplementation improves insulin resistance through suppressed inflammation in obese women with the metabolic syndrome: A randomised controlled trial. Diabetologia.

[75] Harita N., Hayashi T., Sato K.K., Nakamura Y., Yoneda T., Endo G., Kambe H. (2009). Lower Serum Creatinine Is a New Risk Factor of Type 2 Diabetes. Diabetes Care.

[76] Takeuchi M., Imano H., Muraki I., Shimizu Y., Hayama-Terada M., Kitamura A., Okada T., Kiyama M., Iso H. (2018). Serum creatinine levels and risk of incident type 2 diabetes mellitus or dysglycemia in middle-aged Japanese men: A retrospective cohort study. BMJ Open Diabetes Res. Care.

[77] Chutani A., Pande S. (2017). Correlation of serum creatinine and urea with glycemic index and duration of diabetes in Type 1 and Type 2 diabetes mellitus: A comparative study. Natl. J. Physiol. Pharm. Pharmacol..

[78] Carpéné C., Schaak S., Guilbeau-Frugier C., Mercader J., Mialet-Perez J. (2016). High intake of dietary tyramine does not deteriorate glucose handling and does not cause adverse cardiovascular effects in mice. J. Physiol. Biochem..

[79] Visentin V., Marq P., Bour S., Subra C., Prévot D., Morin N., Valet P., Monje M.C., Nepveu F., Carpéné C. (2003). Effect of prolonged treatment with tyramine on glucose tolerance in streptozotocin-induced diabetic rats. J. Physiol. Biochem..

